# The development of a web-based app employing machine learning for delirium prevention in long-term care facilities in South Korea

**DOI:** 10.1186/s12911-022-01966-8

**Published:** 2022-08-17

**Authors:** Kyoung Ja Moon, Chang-Sik Son, Jong-Ha Lee, Mina Park

**Affiliations:** 1 College of Nursing, Keimyung University, 1095 Dalgubeol-daero, Dalseo-gu, Daegu, 42601 South Korea; 2 Division of Intelligent Robots, Daegu Gyeongbuk Institute of Science and Technology (DGIST), 333, Techno jungang-daero, Hyeonpung-eup, Dalseong-gun, Daegu, South Korea; 3 College of Medicine, Keimyung University, 1095 Dalgubeol-daero, Dalseo-gu, Daegu, 42601 South Korea

**Keywords:** Clinical decision support system, Delirium, Long-term care facility, Mobile apps, Rule-based prediction

## Abstract

**Background:**

Long-term care facilities (LCFs) in South Korea have limited knowledge of and capability to care for patients with delirium. They also often lack an electronic medical record system. These barriers hinder systematic approaches to delirium monitoring and intervention. Therefore, this study aims to develop a web-based app for delirium prevention in LCFs and analyse its feasibility and usability.

**Methods:**

The app was developed based on the validity of the AI prediction model algorithm. A total of 173 participants were selected from LCFs to participate in a study to determine the predictive risk factors for delerium.

The app was developed in five phases: (1) the identification of risk factors and preventive intervention strategies from a review of evidence-based literature, (2) the iterative design of the app and components of delirium prevention, (3) the development of a delirium prediction algorithm and cloud platform, (4) a pilot test and validation conducted with 33 patients living in a LCF, and (5) an evaluation of the usability and feasibility of the app, completed by nurses (Main users).

**Results:**

A web-based app was developed to predict high risk of delirium and apply preventive interventions accordingly. Moreover, its validity, usability, and feasibility were confirmed after app development. By employing machine learning, the app can predict the degree of delirium risk and issue a warning alarm. Therefore, it can be used to support clinical decision-making, help initiate the assessment of delirium, and assist in applying preventive interventions.

**Conclusions:**

This web-based app is evidence-based and can be easily mobilised to support care for patients with delirium in LCFs. This app can improve the recognition of delirium and predict the degree of delirium risk, thereby helping develop initiatives for delirium prevention and providing interventions. Moreover, this app can be extended to predict various risk factors of LCF and apply preventive interventions. Its use can ultimately improve patient safety and quality of care.

**Supplementary Information:**

The online version contains supplementary material available at 10.1186/s12911-022-01966-8.

## Background

Delirium, characterised by its sudden onset, causes changes in consciousness, memory, logical reasoning, concentration, and the performance of activities [1]. Its prevalence is 24.6% among patients older than 65 years who are admitted to acute care facilities, 7.9% among those with dementia or recovering from a stroke [2], and 29–31% among inpatients in general wards [3]. The prevalence of postoperative delirium varies widely, from 12 to 51%, depending on the type of surgery [4]. In South Korea, the incidence of delirium is 20% among intensive care unit patients [5], whereas in long-term care facilities (LCFs), the incidence of delirium is only 8.1%. The incidence of delirium that is comorbid with dementia is 39.9% [6].

Delirium increases the mortality rate [4, 6, 7], incidence of falls, presence of pressure ulcers [8, 9], length of hospital stays, degree of medical burden [10–13], rate of chronic cognitive impairment, and likelihood of readmission to LCFs [14–16]. Early detection and preventive interventions using standardised assessment tools [17, 18] are more effective for delirium than are its treatment and management [4, 19]. However, these measures are rarely applied [20, 21]. In addition, it is important to approach delirium intervention with evidence-based multi-components [19, 22, 23].

Older adults admitted to LCFs often have cognitive, visual, or hearing impairments, which place them at risk for delirium [21, 24]. Usually, LCFs in South Korea are not equipped to provide high-level care for delirium patients and do not have an electronic medical record system, as seen in general hospitals [6]. This hinders systematic approaches, including delirium monitoring. Therefore, a system that predicts the onset of delirium and enables the timely administration of preventive interventions is necessary.

A health program using mobile technology that includes delirium prediction, assessment, and prevention intervention according to delirium risk factors, is advantageous as it can be used by various health providers at a low cost [25]. When the clinical decision support system is integrated into mHealth technology, it improves the accuracy of diagnosis and treatment [26, 27]. In addition, implementing such a system on a mobile device offers portability, the possibility of customisation, and convenience [28].

Machine learning uncovers useful information hidden within data and is able to generate an explanatory, predictive, or normative tool based on this information [25, 29]. Of the different types of machine learning algorithms, random forest, artificial neural networks, and support vector machines are widely used in various clinical apps. They have been shown to exhibit high performance in disease diagnosis and prognostic predictions [26, 28, 30–32].

The purpose of this study is to develop a web-based delirium prevention app (Web_DeliPREVENT_4LCF) that uses machine learning to predict the risk of delirium and provide evidence-based interventions for patients in LCFs. It applies a multi-component program and a capitalised mobile clinical decision support system to support health providers working in LCFs. Furthermore, this study assesses the web app’s usability and feasibility relative to its intended use.

## Methods

The Ahituv model [33] was revised and used for app development. Ahituv's model is a basis for the development of the app because the app has the potential to support clinical decision-making [33].

The development process consisted of five phases. The guidelines set forth by the Scottish Intercollegiate Guidelines Network [34] were used to select the evidence-based delirium risk factors and preventive interventions. Iterative design was performed and cloud computing for machine learning to predict the high risk of delirium. The prediction algorithm was validated using the risk factor data of 33 LCF residents and subsequently implemented into the delirium prevention app [35]. The app was then pilot tested, and its usability and feasibility were analysed.

Two nursing professors, one biomedical engineering professor, two machine learning experts, one app development expert, and three clinical field nurses participated in this study to proceed with its five phases. For step-by-step research progress, the related literature was referred to.

### Conceptual framework of the app

The app developed in this study was based on Ahituv’s clinical decision model, an information flow model that involves the observation of a patient, interpretation of a patient's information, drawing of conclusions based on stored clinical knowledge, receipt of clinical advice, and finally, action [36].

**Fig. 1:**
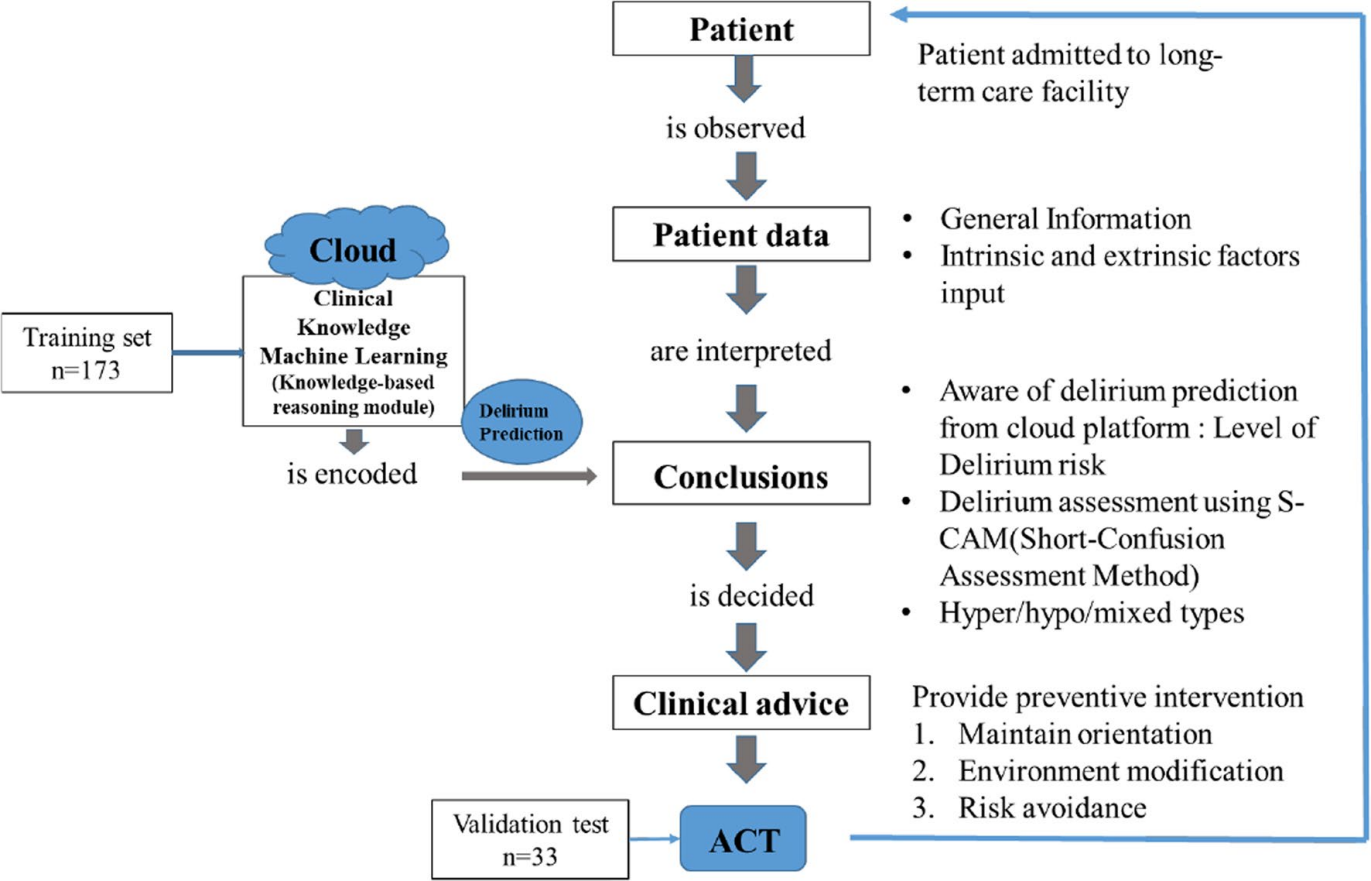
Modified version of the Ahituv model for developing Web_DeliPREVENT_4LC

Web_DeliPREVENT_4LCF consists of four domains: patient data input, prediction results for the input patient’s delirium risk factors, delirium assessment using the Short version of the Confusion Assessment Measure (S-CAM), and application of delirium preventive intervention (Fig. 1).

### Selecting delirium risk factors and non-pharmacological interventions through an evidence-based literature review

Search strategy and eligibility were determined using Scottish Intercollegiate Guidelines Network guidelines [34] and applied to delirium risk factors and preventive interventions in the literature. Individual studies, review articles, and published systematic reviews were searched in MEDLINE, PubMed, CINAHL, Cochrane Library, Embase, PROQuest, KoreaMed, KMbase, and KISS (Korean studies Information Service System) using the terms ‘delirium’, ‘prevention’, ‘intervention’, ‘long-term care’, ‘nursing home’, and ‘mobile apps’. The publication year was set between 2010 and 2018. Various guidelines were searched using the search terms ‘delirium’ and ‘prevention.’

Inclusion criteria included a focus on prevention or intervention of delirium in a long-term care setting and mobile apps. Exclusion criteria included non-English publications (except Korean), study protocols, editorials, commentaries, letters, dissertations, and studies that focused exclusively on paediatrics, stroke, alcohol withdrawal, neurosurgery or trauma patient populations or intensive care unit settings.

After reviewing 1507 titles, 194 abstracts and 74 full texts, 23 studies were selected to be included in the literature review. Two researchers reviewed the studies independently and evaluated them critically using the relevant tools such as SIGN checklist RCT, case control studies, cohort studies, diagnostic studies, systematic reviews, and Critical Appraisal Tool for Cross-Sectional Studies (CAT-CSS).

Regarding delirium risk factors (e.g. age, number of comorbidities, pain, and pain medicine use), 24 factors were selected based on the literature review and a previous study [37]. Orientation, environmental interventions, and early avoidance of the intrinsic and extrinsic risk factors for delirium were selected as multi-components and non-pharmacological interventions for delirium prevention (Text box 1).

**Text box 1:** The four main menus of Web_DeliPREVENT_4LCF

Information: app using tips, general information on delirium (definition, assessment tools, intervention) Risk prediction: select general characteristics and risk factors presented by the patient, implement delirium prediction algorithm on the cloud platform, calculate and present the delirium risk rates Delirium assessment: shows the delirium result after the Short Confusion Assessment Measure questionnaire embedded in the app Multi-components intervention: maintain orientation, environmental modification, risk avoidance	

### Prediction algorithm pilot test and iterative design

The validity of the delirium prediction algorithm was evaluated using explainable machine learning models utilizing data from 33 LCF participants between 10 and 25 August 2020. Machine learning algorithms, such as C4.5, CBA, MCAR, and LEM2, and statistical learning algorithms, such as LR, ANNs, SVMs with three kernel functions, and random forest, were validated by paired Wilcoxon signed-rank tests on both macro -averaged F1 and weighted average F1-measures during the 10-times stratified twofold cross-validation [35]. Furthermore, for a user-friendly design, five nurses working at an LCF participated in evaluating the appropriateness and given comments of the app.

### User validation

The System Usability Scale (SUS), a tool developed by Brooke in 1996 [38] that evaluates the usefulness of apps, was translated into Korean after obtaining permission from the developer. This tool was validated and modified by two professors and clinical experts each in accordance with the purpose of this study (e.g. convenience of using the app, continuous use and usefulness, time-consuming state, use of CAM, delirium prediction, and initiating intervention) and usability and feasibility were assessed using this modified SUS on 32 LCF nurses.

The SUS is a 10-item questionnaire scored on a 5-point Likert scale ranging from 1 (not at all) to 5 (strongly agree). The overall score is calculated by summing the item scores and multiplying the total by 2.5. The score ranges from 0 to 100, with higher scores indicating greater usefulness. A score of 68 or higher is considered above average in terms of usability. At the time of the tool’s development, Cronbach α = 0.85.

## Results

### Development of the Delirium Prediction Algorithm and Cloud Platform

A knowledge-based reasoning module that enables early screening of delirium based on the data collected from a prospective cohort study [6] was conducted with older adults in two LCFs (120-bed and 100-bed) in two cities from October 2016 to March 2017. Predictive performance was assessed with macro-averaged accuracy (71.7%), sensitivity (74.4%), specificity (71.6%), and AUC (73.8%) during a statistical five-fold cross-validation experiment. When compared to three machine learning algorithms—random forest, artificial neural network, and support vector machine with a radial basis kernel function—AUC performance improved by 6.2%, 2.1%, and 3.3% on average, respectively [39].

**Fig. 2:**
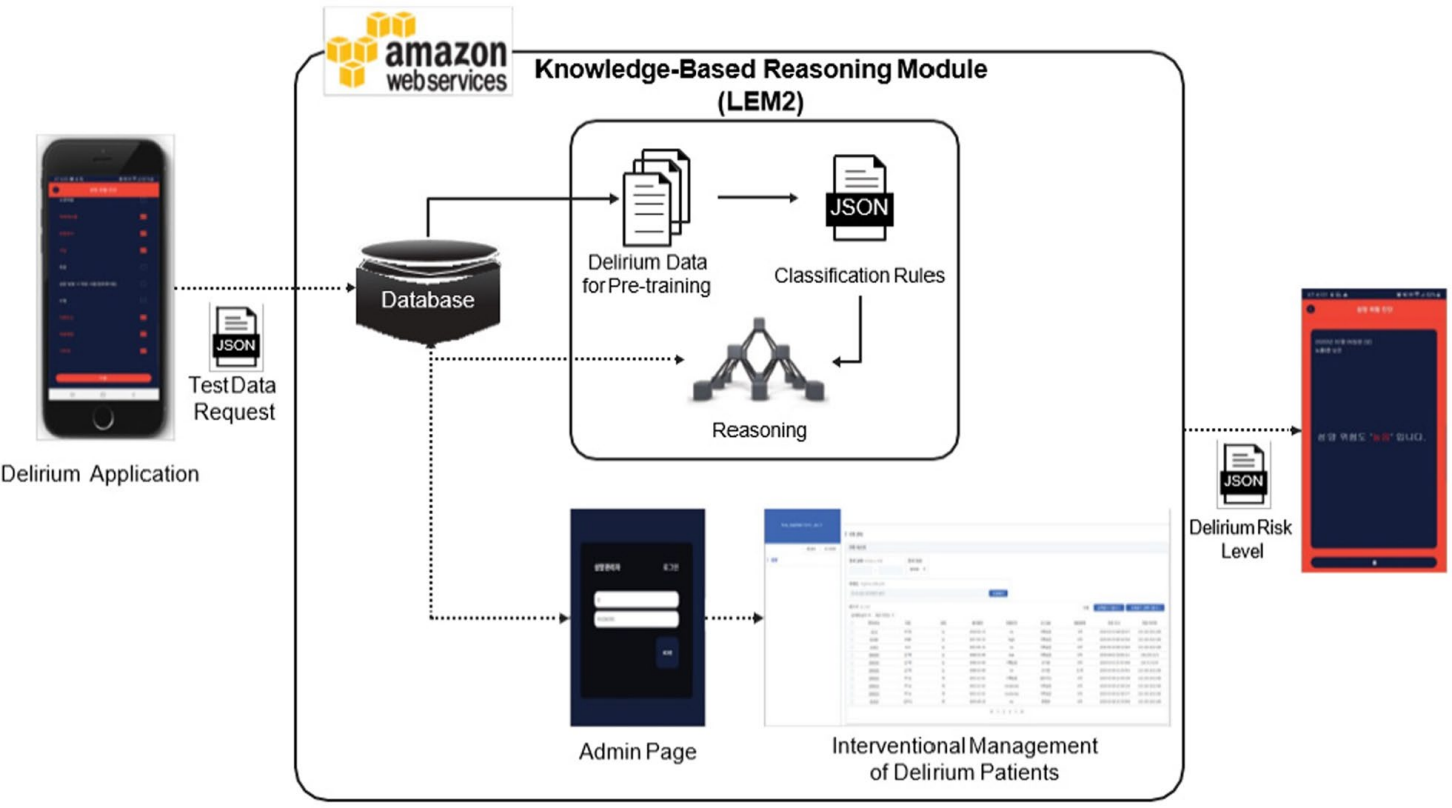
The Web_DeliPREVENT_4LCF Cloud platform

Figure 2 shows the module of the delirium prevention app designed to operate in the Amazon Web Services environment. The knowledge-based reasoning module is divided into delirium classification rules (Additional file 1: Appendix 1) and reasoning processes (Additional file 1: Appendix 2). Additional file 1: Appendix 2 shows the pre-learned knowledge to screen delirium from non-delirium. This is a delirium classification rule extracted by selecting 24 delirium risk factors (Additional file 1: Appendix 3) from the data collected in a prospective cohort study (N = 173) by Moon and Park [6] and using the knowledge-based reasoning method employed by Son et al. [39].

To estimate the risk of delirium, the matching degree between each experimental sample and the delirium classification rules (i.e. the percentage of perfect matching of an experimental sample with the conditions of a rule) was calculated. When only one candidate rule had the highest matching degree, it was determined as the best; if there were two or more candidate rules, then the rule with the highest value of support × confidence value was considered the best. As in Phase 2, when the output (decision) of the best rule was ‘delirium’, the risk for delirium was estimated as low when the support for the rule was smaller than 1.73%, medium when the support ranged from 1.73% to 3.47%, and high when it exceeded 3.47%. Additionally, the structure of reasoning knowledge (delirium classification rules) applied to the delirium prevention app was established in JavaScript Object Notation format to enable easy revision and correction by healthcare providers as well as integration or expansion with knowledge extracted from new delirium data in the future. Installed algorithms upgrade the degree of the risk based on accumulated patient data as well as the continued analysis of the risk factors and level of incidence of delirium among patients.

Cases with ‘YES’ responses for attention deficits in Step 2, at least one YES in Box 1 that combines Steps 1 and 2, and at least one YES in Box 2 that combines Steps 3 and 4, were determined as having delirium (+ ; Additional file 1: Appendix 1). When that was the case, the delirium result was presented along with a warning alarm for delirium. The warning alarm is provided as a screen alarm, sound alarm, vibration alarm, or any combination, for healthcare providers as notifications to check delirium results. In this study, screen and vibration alarms were used.

### Iterative design and composing the components of Web_DeliPREVENT_4LCF

With regard to the app design, Google and Android markets examined to determine current development trends and ascertain whether there were any prototypes or existing delirium applications. Searching keywords were ‘delirium’, ‘applications’, and ‘predictions’ and expert validity achieved in the content.

First, the app provides usage tips, including instructions, recommendations, and information about delirium, such as its definition, the risk factors, and key intervention points. Second, the user is requested to enter the patient’s information, including sex, birth date, diagnosis, severity of delirium, and 10 applicable risk factors (e.g. aged ≥ 65 years, disease severity, abnormal blood urea nitrogen, dehydration, water-electrolyte imbalance, nutritional imbalance, hypoxia, infection, sleep disorder, surgery with general anaesthesia, and oedema). The app then automatically calculates the risk of delirium using the established equation in a percentage and shows whether the patient belongs to the high-, moderate-, or low-risk group. This allows healthcare providers to predict the onset of delirium and pay closer attention to the patient in advance or effectively prevent the sudden onset of delirium. Delirium risk is classified as high, moderate, and low, with the 33.3rd percentile (1.73%) and 66.7th percentile (3.47%) as the cut-off markers. The support values for 23 delirium-related rules (Nos. 1–23) in the ‘IF’ condition and ‘THEN’ decision were used (Additional file 1: Appendix 1).

Next, the type of delirium (hyperactive, hypoactive, or mixed) is assessed among patients testing positive for delirium in the S-CAM. Written permission was obtained from the developer to use S-CAM in the app [35]. The S-CAM consists of four steps. Step 1 includes two questions that check for acute onset and changes in consciousness; Step 2 checks for attention deficits; Step 3 involves a non-systematic thinking assessment, which estimates whether the patient engages in non-systematic or inconsistent thinking; and Step 4 involves the evaluation of changes in the level of consciousness. Consciousness was evaluated as normal, vigilant, lethargic, stuporous, or comatose, and the result is shown as delirium ( +) or no delirium (−) as embedded assessment rule.

Following the assessment, preventive multi-component interventions are given to high-risk patients testing positive for delirium. The preventive multi-components include orientation interventions, environmental interventions, and early avoidance of risk factors. Orientation interventions include repeated orientation such as: using a clock or calendar, using the patient’s name when providing care (as a reminder); placing familiar objects close to the patient, encouraging family members to visit regularly, early abnormality detection, encouraging regular involvement in the activities of daily living, and providing assistive devices to improve visual and hearing abilities, such as eyeglasses and hearing aids.

However, environmental interventions include the following: using indirect lighting and reduced ambient noise to foster an appropriate sleeping environment, keeping the same nurse in the same ward, distinguishing between day and night by using window curtains or blinds, encouraging patients to continue their hobbies (such as listening to music, playing games, and doing hand-knitting), providing or reading newspapers daily, and encouraging meaningful conversations to stimulate memory and reasoning skills.

**Fig. 3:**
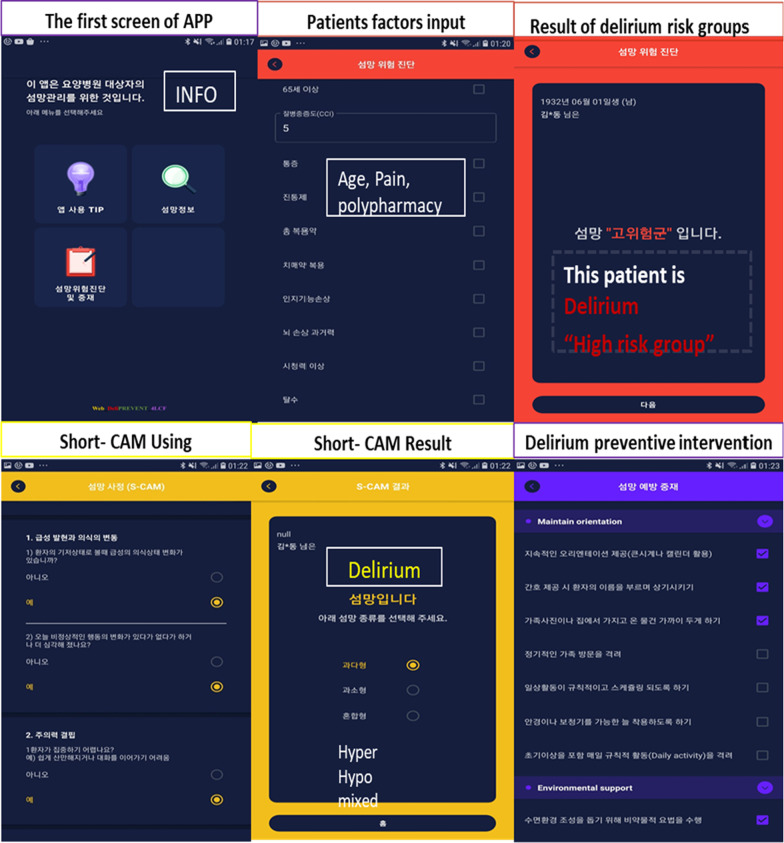
Web_DeliPREVENT_4LCF app screenshot of patient factors input and risk group and delirium assessment results (S-CAM)

The early avoidance of risk factors refers to actions such as: providing an appropriate amount of water and preventing dehydration, pharmacological and non-pharmacological interventions for pain, minimising the use of restraints, a range of active and passive motion exercises, encouraging walking, encouraging drinking water, evaluating nutritional intake, providing non-oral feeding if necessary, the early detection of and intervention for infection, careful use of anticholinergic drugs and opioids and minimising the use of unnecessary drugs, monitoring hypoxia, and preventing constipation, falls, and pressure ulcers. Healthcare providers are asked to mark each intervention item when completed and to calculate the patient’s performance rate. Figure 3 shows a screenshot of the delirium prevention app for patients in LCFs.

### Pilot testing and analysis of delirium prediction validity

To assess the validity of the prediction by the developed app, the Web_DeliPREVENT_4LCF app was applied to a sample of 33 adult (≥ 18 years) inpatients at a LCF for two weeks. In the initial stage of the study, inpatients at a long term care facility in Korea were recorded as adults > 18 years of age. However, in majority of the long-term care facilities, they were older than 65 years of age.

Approval was obtained from K University’s Institutional Review Board. Exclusion criteria included the inability to perform S-CAM owing to psychiatric diagnosis, severe visual or hearing impairment, death, transfer on the day of admission, emergency treatment at the time of delirium assessment, and having other hospital affairs. The experimental cases were collected using the mobile delirium prevention app between 10 and 25 August 2020. These cases were examined through the knowledge-based reasoning module to screen for delirium risk. To validate the delirium prediction, a pilot study was performed using multiple machine learning algorithms, including four rule-mining algorithms (C4.5, CBA, MCAR, and LEM2) and four other statistical learning algorithms (LR, ANNs, SVMs with three kernel functions, and random forest) by paired Wilcoxon signed-rank tests on both macro-averaged F1 and weighted average F1-measures during the 10-times stratified twofold cross-validation. Post analysis, the LEM2 algorithm showed the best prediction performance (macro-averaged F1-measure of 49.35%; weighted average F1-measure of 96.55%), sharply identifying patients at risk of delirium. Pairwise comparisons between predictive powers were observed in independent models, where the LEM2 model had moderate or large effect sizes, between 0.4925 and 0.8766, when compared to the LR, ANN, SVM with RBF, and MCAR models [35].

### Usability and feasibility of the Web_DeliPREVENT_4LCF (Tables 1, 2, and 3)

**Table 1:** General characteristics of participants (N = 32)

Characteristics	Categories	N (%) or Mean ± SD
Sex	Female	32 (100)
Age (years)		45.31 ± 8.26
Education level	Diploma	12 (38)
	Bachelor’s	15 (47)
	> Master’s	5 (16)
Total work experience (years)		16.09 ± 6.55
Long-term care facility work experience (years)		6.56 ± 4.24
Position	Staff nurse	9 (28.1)
	Charge nurse	2 (6)
	Head nurse	19 (59)
	Others	2 (6)
Experience of delirium care	Yes	22 (68.8)
	No	10 (31.3)
Experience of delirium assessment tool use	Yes	1 (3.1)
	No	31 (96.9)
Education experience of delirium care	Yes	25 (78.1)
	No	7 (21.9)
Pathway of delirium care education	Hospital	12 (37.5)
	Nursing school	9 (28.1)
	Self-directory education	1 (3.1)
	Others	3 (9.4)
Self-evaluation of using a smartphone or tablet PC	Very good	4 (12.5)
	Good	4 (12.5)
	Moderate	19 (59.4)
	Poor	5 (15.6)

**Table 2:** Usability score on a 5-point scale by item (N = 32)

Item	Mean ± SD
A personal smartphone is convenient to use the app	4.13 ± 0.87
Wi-Fi is convenient when using the app	4.19 ± 0.86
Personal data are more convenient than public Wi-Fi when using the app	3.62 ± 1.21
The app is easy to use	3.41 ± 0.98
The app is unnecessarily complex	3.06 ± 1.05
I think most people will learn how to use the app very quickly	3.47 ± 0.95
I felt very confident in using the app	3.28 ± 0.77
A lot of learning is required before using the app	2.66 ± 0.83
I would use using the app when caring for a delirium patient	3.91 ± 0.93

**Table 3:** Feasibility score on a 5-point scale by item (N = 32)

Item		N (%) or Mean ± SD
The app is suitable for caring for delirium patients in a long-term care facility		3.72 ± 0.63
The use of CAM^a^ through the app makes it easier to assess delirium patients		3.81 ± 0.69
The delirium prediction result warned about the possibility of developing delirium patients, which led to caution		3.84 ± 0.72
Initiating care for delirium patients was achieved through the results of the app’s delirium prediction and delirium assessment		3.88 ± 0.61
The use of the app made it easy to apply delirium interventions		3.84 ± 0.63
The use of the app has improved the overall knowledge of delirium		3.88 ± 0.71
The app is useful for clinical use		3.78 ± 0.66
I will continue to use this app for delirium intervention in the future		3.66 ± 0.79
Average time taken for one use of the app (minute)	2 <	18 (56.3)
Average number of times to get used to using the app (Count)	3–5	17 (53.1)
Total time it took to get used to using the app (minute)	60	13 (40.6)

^a^Confusion Assessment Method

To evaluate its usability and feasibility, Web_DeliPREVENT_4LCF was approved by the K University Institutional Review Board (IRB No.40525–202,101). Data were collected from 13 to 31 March 2021 for nurses who had been working in LCFs for more than six months. In total, 33 questionnaires were collected. After excluding one questionnaire for having incomplete responses, 32 questionnaires were used for analysis (97% response rate).

The data were analysed using the SPSS WIN 23.0. The general characteristics of the study participants are shown in Table 1, the usability of the app in Table 2, and the feasibility of the app in Table 3.

Regarding the app’s usability, the items stating that a personal smartphone is more convenient than a tablet PC and that it is more convenient to use Wi-Fi than personal data received a high score. The lowest score was given to the item stating that the training for using the app consumes a lot of time.

Regarding the app’s feasibility, the item stating that ‘Initiating care for delirium patients was achieved through the results of the app’s delirium prediction and delirium assessment,’ and that ‘The use of the app has improved the overall knowledge of delirium’ received the highest scores. Particularly, 68.8% of respondents had experience with delirium care, but only 3.1% felt that they would not actively use the tools during delirium care.

## Discussion

This study describes the development of an Android mobile app for delirium prevention among patients at LCFs: Web_DeliPREVENT_4LCF. When patient information and delirium risk factors were entered into the app through a web server connection, the app predicted the risk of delirium (high, moderate, or low) based on a knowledge-based reasoning module. Through the app, healthcare providers can be notified of patients’ risk levels for delirium and assess delirium accordingly using the S-CAM. The app then shows the delirium prevention intervention screen and instructs the provider to apply multi-component interventions.

This app could serve to help LCFs when the awareness of delirium is low and when the facility is poorly equipped for delirium assessment or preventive interventions compared to larger hospitals [6, 39]. Furthermore, by reflecting the importance of real-time information at the point of care in the practice of evidence-based intervention, this app also provides effective communication among health professionals [40, 41].

Web_DeliPREVENT_4LCF was designed to predict current delirium risk levels based on up-to-date patient factors entered into the system, to assess delirium using a validated delirium assessment tool, and to help healthcare providers apply multi-component preventive interventions. Moreover, the entered app data are connected to the web server; thus, the app uses the prediction algorithm installed online to calculate patients’ delirium risks as percentages (%). The predictive accuracy increases as data accumulate (i.e. as higher numbers of patients and risk factors are entered into the system) [39].

When considering the poor outcomes of delirium among patients within LCFs [6, 42], the use of Web_DeliPREVENT_4LCF increases awareness of delirium among healthcare providers and presents a prediction of patients’ risks, thereby enabling healthcare providers to begin assessing delirium and proper intervention immediately and accurately. Thus, Web_DeliPREVENT_4LCF can be regarded as a mobile clinical decision support system that helps health providers make clinical judgements about delirium care, rather than the concept of a mobile health app used for patient or caregiver education. In addition, the use of the application may improve the quality of care for elderly patients in LCFs in Korea, where assessment and preventive nursing are sledom performed due to low awareness of delirium among healthcare providers. LCFs typically have a somewhat lower CDSS function compared to general hospitals [43, 44], so this is expected to increase the overall care level in LCFs. In future studies, it would be beneficial to develop similar predictive programs for falls, bedsores, hospital associated infections, and other common occurrences in order to improve the quality of care in LCFs [43, 45].

Regarding the tool’s usability and feasibility, surveyed users responded that Web_DeliPREVENT_4LCF improved their knowledge of delirium, raised awareness about the onset of delirium, and facilitated the app of delirium intervention. Most of the nurses had received education on delirium care, and they actually perform delirium care in the LCFs, but the rate of delirium assessment conducted using a standardised tool was remarkably low. Therefore, the use of Web_DeliPREVENT_4LCF is expected to help improve the quality of delirium care, as standardised delirium assessment tools can be easily applied, and this is thought to be helpful for early detection and intervention [15, 42], which is the goal of delirium management.

## Limitations

This app can be used on a smartphone, tablet or PC by healthcare providers, but one limitation of the design was that this app is operated over the Internet. Thus, it may be of limited use when Internet connections are unstable, and it may not yet be feasible in all long-term care hospitals.

A second limitation was that the risk factors of patients must be continuously monitored, periodic updates are not performed automatically, and it is difficult to contact an external technician.

The environment for the patients from whom the random forest data were collected in a previous study [6] (which were the data used for developing the algorithm) differed from that of the patients enrolled in the pilot study. It can be assumed that the current COVID-19 pandemic had some influence; thus, subsequent studies should recruit a larger study population and continuously apply and analyse prediction algorithms based on new risk factors in this population.

Another limitation was patient information. When entering patient information in the app, only the surname is entered as the patient’s ID. Additionally, when being synchronised with Amazon Web Services, patients’ IDs were encoded again per the secure coding standard. Nevertheless, a fundamental problem, the security of cloud-uploaded data, is socially present; therefore, this should be continuously complemented and updated through app and version upgrades.

Although this study was conducted in such a manner that any potential selection bias would be minimised, the possibility of selection bias occurring should be considered with respect to the app contents and pilot testing. Although the usefulness of the app, as well as its ease of use have been confirmed, there may still be limitations in the generalisation of our findings to the wider population due to the small sample size.

## Conclusions

Despite the high incidence of delirium among patients at LCFs, awareness of delirium is still low, and appropriate assessments and interventions are not actively performed in long-term care hospitals. Web_DeliPREVENT_4LCF app presents healthcare providers with timely and convenient predictions of patients’ delirium risks, assisting them in assessing delirium with a validated tool and administering delirium prevention interventions. This app could ultimately contribute to patient safety and quality of care, including lower mortality rates, reduced durations of hospital stay, and lower medical costs.

## Supplementary Information

**Additional file 1**.** Appendix 1**. Development of delirium prediction algorithm utilizing a knowledge-based reasoning module. 1-1. Data characteristics. 1-2. Classification rules to identifying the delirium from non-delirium patients. 1-3. Reasoning procedure to estimate the risk level of delirium.

## Data Availability

Not applicable.

## Abbreviations

LCFs: Long-term care facilities
S-CAM: Short version of the confusion assessment measure
KISS: Korean studies information service system
CAT-CSS: Critical appraisal tool for cross-sectional studies
SUS: System usability scale
