# Supplementary material for: The development of a web-based app employing machine learning for delirium prevention in long-term care facilities in South Korea
Source: BMC Med Inform Decis Mak. 2022 Aug 17;22:220. doi: 10.1186/s12911-022-01966-8 (PMC9383654; doi:10.1186/s12911-022-01966-8)
Supplement: Supplementary file 1 — Additional file 1. Appendix 1. Development of delirium prediction algorithm utilizing a knowledge-based reasoning module. 1-1. Data characteristics. 1-2. Classification rules to identifying the delirium from non-delirium patients. 1-3. Reasoning procedure to estimate the risk level of delirium. [file 12911_2022_1966_MOESM1_ESM.docx]

Appendix 1. Development of delirium prediction algorithm utilizing a knowledge-based reasoning module

1-1 Data characteristics

| Variables | Delirium (N=83) | Non-Delirium (N=90) |
| --- | --- | --- |
| Older | Yes: 80(96.4) | Yes: 62 (68.9) |
| Severity | 6.66±2.99 | 3.11±3.13 |
| Ache | Yes: 24(28.9) | Yes: 34(37.8) |
| Analgesic | Yes: 42(50.6) | Yes: 47(52.2) |
| Medication | Yes: 83(100.0) | Yes: 88(97.8) |
| Dementia_Drug_Use | Yes: 40(48.2) | Yes: 35(38.9) |
| Cognitive_Damage | Yes: 50(60.2) | Yes: 32(35.6) |
| Brain_Damage_Anamnesis | Yes: 8(9.6) | Yes: 8(8.9) |
| Vision_Hearing_Impairment | Yes: 9(10.8) | Yes: 11(12.2) |
| Dewatering | Yes: 3(3.6) | Yes: 3(3.3) |
| Innutrition | Yes: 39(47.0) | Yes: 11(12.2) |
| Electrolyte_Imbalance | Yes: 7(8.4) | Yes: 1(1.1) |
| Surgery | Yes: 7(8.4) | Yes: 3(3.3) |
| Infection | Yes: 11(13.3) | Yes: 11(12.2) |
| Sleep_Deprivation | Yes: 30(36.1) | Yes: 11(12.2) |
| Depressant | Yes: 1(1.2) | Yes: 1(1.1) |
| Stationary | Yes: 3(3.6) | Yes: 3(3.3) |
| Fall | Yes: 26(31.3) | Yes: 49(54.4) |
| Decubital | Yes: 25(30.1) | Yes: 9(10.0) |
| Haloperidol | Yes: 15(18.1) | Yes: 3(3.3) |
| Transfuse | Yes: 0(0.0) | Yes: 0(0.0) |
| Catheterization | Yes: 10(12.0) | Yes: 4(4.4) |
| Gavage | Yes: 11(13.3) | Yes: 4(4.4) |
| Diaper | Yes: 50(60.2) | Yes: 23(25.6) |

1-2 Classification rules to identifying the delirium from non-delirium patients

| No | IF | THEN | Statistical Evidence | |
| --- | --- | --- | --- | --- |
|  | Conditions | Decision | Support(%) | Confidence |
| 1 | Cognitive_Damage is YES AND Sleep_Deprivation is YES AND Decubital is YES | Delirium | 5.2 | 1.0 |
| 2 | Sleep_Deprivation is YES AND Haloperidol is YES | Delirium | 5.2 | 1.0 |
| 3 | Diaper is YES AND Severity ≤ 4.5 AND Cognitive_Damage is YES AND Sleep_Deprivation is YES | Delirium | 1.7 | 1.0 |
| 4 | Cognitive_Damage is NO AND Fall is NO AND Innutrition is YES AND Ache is YES | Delirium | 3.5 | 1.0 |
| 5 | Ache is NO AND Innutrition is YES AND Sleep_Deprivation is NO AND Diaper is YES AND Dementia_Drug_Use is YES | Delirium | 2.3 | 1.0 |
| 6 | Severity > 4.5 AND Dementia_Drug_Use is NO AND Sleep_Deprivation is YES | Delirium | 4.6 | 1.0 |
| 7 | Sleep_Deprivation is NO AND Severity > 4.5 AND Fall is NO AND Diaper is YES AND Cognitive_Damage is NO | Delirium | 5.2 | 1.0 |
| 8 | Sleep_Deprivation is NO AND Vision_Hearing_Impairment is NO AND Fall is YES AND Cognitive_Damage is YES AND Analgesic is NO AND Ache is NO | Delirium | 2.9 | 1.0 |
| 9 | Severity > 4.5 AND Fall is NO AND Dementia_Drug_Use is NO AND Diaper is YES | Delirium | 13.3 | 1.0 |
| 10 | Severity > 4.5 AND Ache is YES AND Analgesic is YES AND Fall is YES | Delirium | 1.7 | 1.0 |
| 11 | Severity > 4.5 AND Analgesic is YES AND Sleep_Deprivation is YES | Delirium | 5.8 | 1.0 |
| 12 | Infection is NO AND Diaper is NO AND Innutrition is YES AND Ache is NO AND Dementia_Drug_Use is NO | Delirium | 2.3 | 1.0 |
| 13 | Ache is YES AND Catheterization is NO AND Gavage is YES | Delirium | 1.2 | 1.0 |
| 14 | Analgesic is YES AND Stationary is YES | Delirium | 1.2 | 1.0 |
| 15 | Analgesic is YES AND Catheterization is YES | Delirium | 3.5 | 1.0 |
| 16 | Decubital is NO AND Sleep_Deprivation is NO AND Analgesic is NO AND Diaper is NO AND Fall is YES AND Cognitive_Damage is YES | Delirium | 1.7 | 1.0 |
| 17 | Dementia_Drug_Use is YES AND Infection is YES | Delirium | 1.2 | 1.0 |
| 18 | Diaper is NO AND Innutrition is YES AND Sleep_Deprivation is YES | Delirium | 2.3 | 1.0 |
| 19 | Sleep_Deprivation is YES AND Surgery is YES | Delirium | 0.6 | 1.0 |
| 20 | Dewatering is NO AND Cognitive_Damage is NO AND Dementia_Drug_Use is YES AND Fall is NO AND Sleep_Deprivation is NO AND Analgesic is NO | Delirium | 1.2 | 1.0 |
| 21 | Ache is YES AND Cognitive_Damage is NO AND Decubital is YES | Delirium | 1.7 | 1.0 |
| 22 | Infection is NO AND Vision_Hearing_Impairment is NO AND Haloperidol is YES | Delirium | 5.8 | 1.0 |
| 23 | Ache is YES AND Dementia_Drug_Use is YES AND Cognitive_Damage is NO AND Severity > 4.5 | Delirium | 2.3 | 1.0 |
| 24 | Sleep_Deprivation is NO AND Severity ≤ 4.5 AND Older is NO | Non-Delirium | 13.9 | 1.0 |
| 25 | Stationary is NO AND Infection is NO AND Innutrition is NO AND Sleep_Deprivation is NO AND Diaper is NO AND Cognitive_Damage is NO AND Dementia_Drug_Use is NO | Non-Delirium | 15.6 | 1.0 |
| 26 | Cognitive_Damage is YES AND Diaper is NO AND Dementia_Drug_Use is NO AND Analgesic is YES | Non-Delirium | 2.3 | 1.0 |
| 27 | Fall is NO AND Cognitive_Damage is YES AND Diaper is NO AND Innutrition is NO AND Ache is YES | Non-Delirium | 1.2 | 1.0 |
| 28 | Haloperidol is NO AND Dementia_Drug_Use is YES AND Analgesic is NO AND Innutrition is NO AND Severity > 4.5 | Non-Delirium | 2.9 | 1.0 |
| 29 | Ache is NO AND Analgesic is YES AND Severity ≤ 4.5 | Non-Delirium | 7.5 | 1.0 |
| 30 | Vision_Hearing_Impairment is NO AND Dementia_Drug_Use is YES AND Diaper is NO AND Severity ≤ 4.5 | Non-Delirium | 3.5 | 1.0 |
| 31 | Sleep_Deprivation is NO AND Innutrition is YES AND Ache is NO AND Diaper is NO AND Dementia_Drug_Use is YES AND Analgesic is YES | Non-Delirium | 1.2 | 1.0 |
| 32 | Gavage is NO AND Sleep_Deprivation is NO AND Innutrition is YES AND Cognitive_Damage is YES AND Infection is NO AND Dementia_Drug_Use is YES AND Ache is YES | Non-Delirium | 1.2 | 1.0 |
| 33 | Fall is NO AND Analgesic is NO AND Cognitive_Damage is YES AND Innutrition is YES AND Diaper is NO | Non-Delirium | 1.2 | 1.0 |
| 34 | Fall is NO AND Cognitive_Damage is NO AND Diaper is NO AND Infection is YES | Non-Delirium | 1.7 | 1.0 |
| 35 | Decubital is NO AND Severity ≤ 4.5 AND Catheterization is YES | Non-Delirium | 1.2 | 1.0 |
| 36 | Severity ≤ 4.5 AND Haloperidol is NO AND Sleep_Deprivation is YES AND Vision_Hearing_Impairment is NO AND Diaper is NO | Non-Delirium | 4.0 | 1.0 |
| 37 | Innutrition is NO AND Catheterization is NO AND Diaper is YES AND Vision_Hearing_Impairment is YES | Non-Delirium | 1.2 | 1.0 |
| 38 | Dementia_Drug_Use is YES AND Decubital is YES AND Cognitive_Damage is NO | Non-Delirium | 1.7 | 1.0 |
| 39 | Dementia_Drug_Use is YES AND Haloperidol is NO AND Dewatering is YES | Non-Delirium | 1.7 | 1.0 |
| 40 | Decubital is YES AND Depressant is YES | Non-Delirium | 0.6 | 1.0 |
| 41 | Ache is NO AND Dementia_Drug_Use is YES AND Innutrition is NO AND Surgery is YES | Non-Delirium | 0.6 | 1.0 |
| 42 | Diaper is YES AND Fall is YES AND Sleep_Deprivation is NO AND Brain_Damage_Anamnesis is YES | Non-Delirium | 0.6 | 1.0 |
| 43 | Analgesic is YES AND Decubital is NO AND Dementia_Drug_Use is NO AND Haloperidol is NO AND Sleep_Deprivation is YES | Non-Delirium | 2.3 | 1.0 |
| 44 | Haloperidol is YES AND Vision_Hearing_Impairment is YES | Non-Delirium | 1.2 | 1.0 |
| 45 | Fall is YES AND Cognitive_Damage is YES AND Analgesic is YES AND Dementia_Drug_Use is NO | Non-Delirium | 2.3 | 1.0 |

1-3. Reasoning procedure to estimate the risk level of delirium

| **Input**: Given a set of delirium rules *R*, and a test sample *t*  **Output**: One of ‘high-risk’ or ‘medium-risk’ or ‘low-risk’ if delirium; ‘no risk’ otherwise.   1. **For** each rule *r_i_* in *R* **Do** 2. Calculate the matching degree, i.e., the ratio that correctly match *r_i_*’s conditions in a test sample *t*. 3. Find the candidate rules with the largest matching degree 4. if the candidate includes only one rule, it is defined as a best, 5. otherwise, in the candidate, a rule with the largest product value of rule support and confidence is defined as the best. 6. **End For** 7. if the decision of best rule is ‘non-delirum’, return ‘no risk’, 8. if the best rule’s support is less than 1.73 percent, return ‘low-risk’, 9. if the support is between 1.73 and 3.47 percent, return ‘medium-risk’, 10. otherwise, return ‘high-risk’. |
| --- |
